# Acoustic Metamaterial Nanogenerator for Multi-Band Sound Insulation and Acoustic–Electric Conversion

**DOI:** 10.3390/s25216693

**Published:** 2025-11-02

**Authors:** Xinwu Liang, Ming Yuan

**Affiliations:** 1 College of Automation, Nanjing University of Posts and Telecommunications, Nanjing 210023, China; 1023051209@njupt.edu.cn; 2 State Key Laboratory of Flexible Electronics, College of Automation, Nanjing University of Posts and Telecommunications, Nanjing 210023, China

**Keywords:** triboelectric nanogenerator, sound insulation, acoustic–electric conversion, multiple frequency bands, acoustic metamaterial

## Abstract

Controlling low-frequency noise and achieving multi-band sound insulation remain significant challenges and have long been hot topics in industrial research. This study introduces a novel multifunctional device based on the principles of acoustic metamaterials, which not only offers high-performance sound insulation but also converts low-frequency acoustic energy into electrical energy. Through an innovative design featuring multiple local resonance design, the proposed device effectively mitigates the impact of pre-tension on the membrane, while enabling efficient multi-band sound insulation that can be finely tuned by adjusting structural parameters. Experimental results demonstrate that the device achieves a maximum sound insulation of 40 dB and an average sound insulation exceeding 25 dB within the 1000 Hz frequency range. Moreover, by utilizing its local resonance property, a triboelectric nanogenerator (TENG) is specifically designed for low-frequency acoustic–electric conversion, maintaining high performance low-frequency sound insulation while simultaneously powering small scale electronic devices. This work provides a promising approach for multi-band sound insulation and low-frequency acoustic–electric conversion, offering broad potential for industrial applications.

## 1. Introduction

In modern society, noise pollution has become pervasive and represents an urgent challenge owing to its widespread and profound detrimental effects [1,2]. Strategies for noise control generally fall into three categories: controlling the source, the transmission path, or the receiver side [3]. In practice, noise generation is often inevitable, and it remains difficult to fully mitigate noise either at the source or at the receiving side. Consequently, regulating the transmission path through sound insulation has emerged as a practical and effective solution [4]. However, in the low-frequency regime, conventional sound insulation relies on materials with high mass density, which conflicts with the increasing demand for lightweight design in modern equipment [5].

In recent years, the emergence of acoustic metamaterials has opened new avenues for achieving efficient sound insulation at low frequencies [6]. Unlike conventional homogeneous materials, artificial acoustic metamaterials possess subwavelength features and lightweight characteristics, while enabling effective manipulation of low-frequency sound waves [7,8]. Their ability to realize extraordinary equivalent mass properties allows them to exhibit superior sound insulation performance, fundamentally distinct from that of traditional homogeneous media [9]. Among them, membrane-type acoustic metamaterials (MAMs) are particularly attractive due to their compact geometry, lightweight design, and excellent capability to suppress sound transmission [10]. Conventional MAM structures typically employ a tensioned membrane with attached mass blocks, thereby inducing local resonance characteristics at specific frequencies [11,12].

The sound insulation performance of membrane-type acoustic metamaterials primarily depends on the resonant interaction between the membrane and associated auxiliary structures [13]. The sound insulation properties of MAM can be modified by adjusting the configuration and dimensions of the mass blocks, as well as by altering the level of membrane tensions [14,15,16,17]. However, for classical MAMs, achieving a broad sound insulation frequency band or multiple sound insulation peaks within the low-frequency range remains challenging through the adjustment of conventional structural parameters alone.

Hence, researchers have proposed integrating membrane-type sound insulation metamaterials with alternative sound insulation technologies to enhance their performance [18]. For example, Langfeldt et al. [19] actively controlled the effective surface mass density by adjusting the pressure applied by an electrodynamic actuator to the MAM. Li et al. [20] designed a sound insulation structure that integrates a double-layer membrane with a Helmholtz cavity, enabling the generation of multiple sound transmission loss peaks. Additionally, Zhang et al. [21] incorporated a MAM into a conventional double-wall system to form a three-layered acoustic barrier, thereby improving sound insulation performance at resonance dips through the use of porous materials. Nevertheless, these approaches tend to increase both the volume and mass of the device, thereby compromising the lightweight advantages typically associated with MAMs [22].

Moreover, sound energy represents a ubiquitous form of mechanical energy present in the environment [23,24]. During its propagation, sound energy is typically absorbed, insulated, or dissipated by the surrounding medium [25,26]. If this sound energy can be effectively concentrated, amplified, and harvested, it may facilitate the conversion of mechanical energy into electrical energy for efficient management. This advancement could promote the development of zero-power Internet of Things (IoT) applications [27,28,29,30] and hold significant engineering application value. Compared to traditional acoustic–electric conversion methods, such as electromagnetic, piezoelectric approaches, triboelectric nanogenerators (TENGs) offer a diverse range of material options. They can be seamlessly integrated with multiple functionalities and demonstrate high efficiency in harvesting low-frequency and weak mechanical energy. As a result, low-power devices can be powered without batteries through acoustic-to-electric energy conversion [31,32,33].

For example, Yuan et al. [34] proposed a triboelectric nanogenerator based on a conical Helmholtz resonator for acoustic–electric conversion, which is capable of powering sensors for continuous temperature and humidity monitoring. This illustrates an effective approach to utilizing acoustic energy for powering IoT sensor devices. Xiao et al. [35] integrated a conical energy concentrator at the sound inlet and developed a TENG based on a composite quarter-wavelength resonator to enhance electrical output, thereby enabling it to power small devices such as distributed sensor nodes. Yuan et al. [36] introduced a tympanic membrane metamaterial-inspired multifunctional low-frequency acoustic TENG with effective low-frequency sound absorption, which utilizes acoustic energy for self-powered wireless temperature sensing and acoustic communication.

It is evident that TENG-based acoustic–electric conversion systems frequently depend on specific energy conversion components, such as resonators. In addition, numerous systems for acoustic-vibration energy conversion have been developed using film, spring, and mass blocks to construct a resonant system. For instance, Ju et al. [37] designed a flexible amorphous CCTO thin-film energy harvester, fabricated on a plastic substrate, leverages its inherent reliable flexibility to ensure efficient electromechanical energy conversion. Feng et al. [38] proposed a cascaded structure integrating PVDF films and a PDMS-silicon resonant structure; this design achieves multimodal resonance, enabling vibration energy harvesting at the low frequency of 2 Hz. Lee et al. [39] optimized CNF/PVA films and paired them with an innovative cymbal structure—this configuration generates large in-plane strain to enhance the film’s piezoelectric effect, thereby realizing superior vibration energy harvesting.

However, in current acoustic–electric conversion frameworks, resonators predominantly serve the purpose of energy conversion alone; their structural design often neglects to incorporate additional functionalities, which may result in a waste of space resources. Integrating acoustic-to-electric energy conversion with sound insulation devices to achieve multifunctionality warrants sufficient attention.

To address these gaps, the creation of a multifunctional device capable of efficient sound insulation across multiple frequency bands, while also facilitating low-frequency acoustic–electrical conversion, is the central focus of this study. To this end, we introduce a novel metamaterial triboelectric nanogenerator that integrates multi-band sound insulation and acoustic–electrical conversion, referred to as the Multi-Band Sound Insulation Triboelectric Nanogenerator (MBSI-TENG).

For the dynamic component, we propose an innovative planar spring system, which is covered by membranes and augmented with additional mass blocks. This design is inspired by the dispersed petal structure and incorporates a multi-local resonance system that enhances sound insulation performance across multiple frequency bands. Under excitation at a specific frequency, the centrally curved structure induces piston motion to generate contact-separation with the elastic silicone membrane, and the device further exhibits this frequency-specific behavior to produce a corresponding electrical signal, fully demonstrating its promising application potential in self-powered sensing. This mechanism not only preserves sound insulation performance but also enables low-frequency acoustic–electrical conversion.

## 2. Principle and Design of the MBSI-TENG

**Figure 1:**
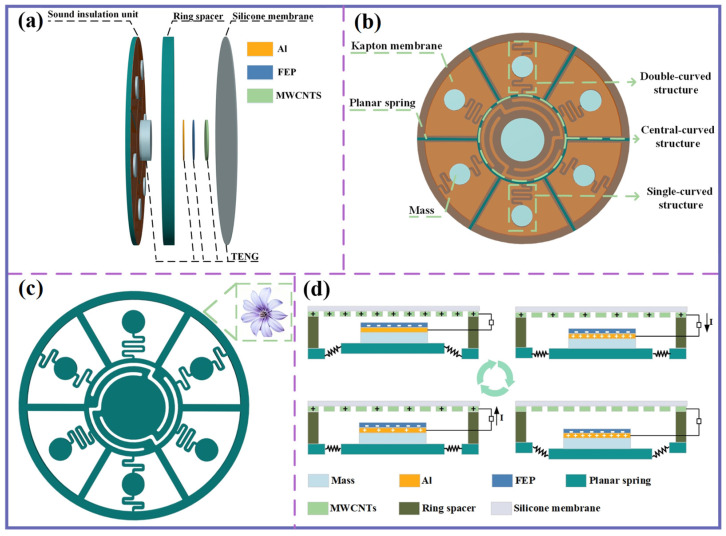
Schematic diagram of the MBSI-TENG. (**a**) Geometric structure of MBSI-TENG. (**b**) Different regions of the sound insulation unit. (**c**) Schematic diagram of the planar spring. (**d**) The working mechanism of MBSI-TENG.

As shown in Figure 1a, the MBSI-TENG consists of a sound insulation unit and a triboelectric nanogenerator. The sound insulation unit, serving as the core component, comprises mass blocks, Kapton membranes, and a planar spring, as depicted in the front view in Figure 1b. Instead of using a single continuous membrane, independent membranes are employed to cover distinct structural regions. This design not only accommodates the differing geometries of the underlying structures but also facilitates membrane replacement during maintenance. Specifically, the central region is covered with a circular membrane, while each of the six surrounding regions is equipped with a fan-shaped membrane. Small gaps are intentionally maintained between adjacent membranes to allow for the application of adhesive, ensuring secure attachment.

The planar spring, with a diameter of 100 mm and a thickness of 2 mm, is strategically positioned at the base of the sound insulation unit to support the Kapton membranes, each of which carries a mass block. Inspired by the morphology of flower pistils, the planar spring features a decentralized structural design. The central receptacle represents the pistil core, while the surrounding “petals” mimic the distributed arrangement of peripheral elements (Figure 1c). This bioinspired configuration introduces intervals between regions, segmenting the spring into seven distinct sections and enriching vibrational modes. Polylactic acid (PLA) is selected as the spring material due to its mechanical flexibility and ease of processing.

Through this innovative structural design, the sound insulation unit integrates three types of localized resonance structures: a central-curved structure, a single-curved structure, and a double-curved structure. Each of these structures consists of a base planar spring, a Kapton membrane, and an attached mass block.

The single-curved structure resembles a cantilever beam. However, unlike a conventional cantilever, its beam length is extended by incorporating a spiral geometry, significantly reducing the stiffness coefficient within the confined space. In contrast, the double-curved structure features two spiral beams, each anchored separately to the outer frame and the central-curved structure. This design enables resonance modes that differ from those of the single-curved structure. Under external acoustic excitation, the central-curved, single-curved, and double-curved structures oscillate at their respective local resonance frequencies.

Kapton membranes, composed of polyimide (PI), are chosen for their superior performance compared to conventional membrane materials such as PET and PTFE [40]. In particular, Kapton offers excellent mechanical strength, high thermal stability, and strong radiation resistance, making it highly suitable for sound insulation applications in harsh environmental conditions [41,42].

To allow for flexible tuning of the resonant frequencies, mass blocks of varying sizes are utilized. The mass block assembly consists of a central block mounted on the central cylindrical disk, with six peripheral blocks positioned on small cylindrical disks surrounding the planar spring, following the attachment of the Kapton membranes. A summary of the material properties relevant to the MBSI-TENG is provided in Table 1.

To achieve acoustic-to-electric conversion through contact electrification, the device developed in this study exploits pronounced local resonance effects in the low-frequency regime, offering an effective strategy for acoustic–electric conversion. Among the three structural configurations, the central-curved structure provides a larger contact area and larger displacement amplitude compared to the single- and double-curved structures. Furthermore, its motion is more uniform, making it particularly suitable for TENG integration.

As shown in Figure 1d, a contact-separation TENG is integrated into the central-curved structure and mounted on the front side of the sound insulation unit. The negative electrode consists of a high-performance fluorinated ethylene propylene (FEP) film, which ensures efficient charge collection. Due to the intrinsic insulating properties of FEP, an aluminum foil layer is introduced as a conductive current collector, enhancing charge transport and facilitating the effective harvesting of negative charges generated on the FEP surface through friction. This negative electrode assembly is then securely attached to the central mass of the central-curved structure.

For the positive electrode, an elastic silicone membrane is used as the substrate, with multi-walled carbon nanotubes (MWCNTs) uniformly deposited on its back surface. The MWCNTs layer serves as the active positive electrode material, benefiting from its large specific surface area, tubular morphology, and superior electrical conductivity. These properties collectively facilitate efficient charge transport and significantly enhance the power generation performance of the TENG.

Under continuous external acoustic excitation, the elastic silicone membrane undergoes sustained vertical oscillations. Meanwhile, the rebound effect of the planar spring drives the central-curved structure into reciprocating motion, causing the central mass block to periodically contact and separate from the silicone membrane. This cyclic process enables repeated contact and separation between the positive and negative electrode materials of the TENG, generating electricity.

## 3. Sound Insulation Analysis of the MBSI-TENG

### 3.1. Sound Insulation Mechanism

**Figure 2:**
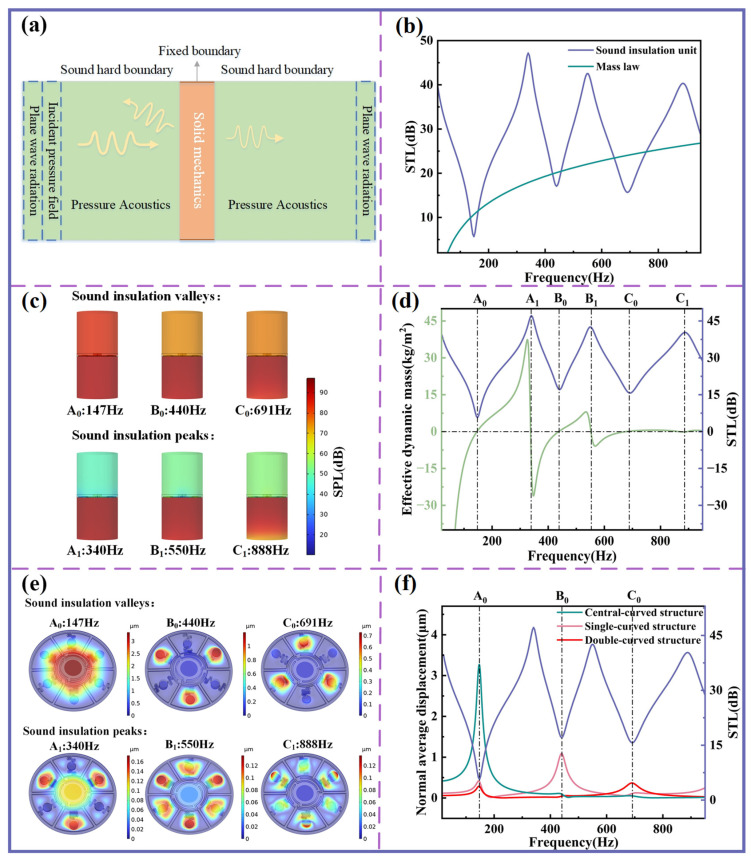
Analysis of sound insulation mechanism. (**a**) Schematic diagram of a numerical acoustic model. (**b**) STL comparison between sound insulation unit and mass law. (**c**) SPL comparison at the peak and valley frequencies. (**d**) Effective dynamic mass and STL curve of the sound insulation unit. (**e**) Modes at the peak and valley of the STL curve. (**f**) Correspondence between the normal displacement of different structures and STL.

In this study, the sound insulation unit integrates three distinct types of local resonance structures. To investigate its sound insulation characteristics, finite element method (FEM) simulations are conducted, with the corresponding physical field model shown in Figure 2a. The model couples two domains: pressure acoustics and solid mechanics domains. The planar spring, mass blocks, Kapton membranes, and ring spacer are assigned to the solid mechanics domain, while the surrounding mediums are modeled as air fluid within the pressure acoustics module.

The incident plane wave’s sound pressure is *p_i_* and the average transmitted sound pressure is *p_ref_*. The sound transmission loss (STL) is calculated as:

(1) $$STL=20{log}_{10}\frac{\left|p_{i}\right|}{\left|p_{ref}\right|}$$

The STL curve calculated from FEM analysis is presented in Figure 2b. For comparison, the areal density of the sound insulation unit is 2.9 kg/m^2^, and the corresponding STL curve predicted by the mass law is also provided. The results show that within the 20–1000 Hz frequency range, three distinct insulation peaks appear at 340, 550, and 888 Hz, accompanied by sound insulation valleys at 147, 440, and 691 Hz.

Figure 2c illustrates the sound pressure level (SPL) distributions at these peak and valley frequencies. At the insulation peaks, the STL values exceed 40 dB, indicating that the unit exhibits excellent sound insulation performance, effectively reflecting most incident acoustic energy. When compared with the STL curve predicted by the mass law, the sound insulation unit demonstrates superior performance across most frequency bands, with the exception of the valleys, where the performance is slightly reduced. Nevertheless, even at the valleys (440 Hz and 691 Hz), the STL remains above 15 dB, highlighting the unit’s robust sound insulation capability across a broad frequency range.

To further elucidate the sound insulation mechanism at different frequencies, the effective dynamic mass *m_dynamic_* of the unit is calculated as:

(2) $$m_{dynamic}=\frac{<∆p>}{<\ddot{\omega }>}$$

where <∆p> is the averaged pressure difference across the sound insulation unit, representing the effective excitation force, and $<\ddot{\omega }>$ denotes the average acceleration of the sound insulation unit. The relationship between the effective dynamic mass and the STL is shown in Figure 2c. For clarity, the three valleys in the STL curve are labeled A_0_ (147 Hz), B_0_ (440 Hz), and C_0_ (691 Hz), while the corresponding peaks are labeled A_1_ (340 Hz), B_1_ (550 Hz), and C_1_ (888 Hz), as marked in the diagram.

The variation in the effective dynamic mass reflects the ability of the sound insulation unit to attenuate acoustic waves at different frequencies. At the sound insulation valley points (A_0_, B_0_, C_0_), the effective dynamic mass exhibits smooth transitions with small values, allowing acoustic waves to transmit through the device easily. In contrast, at the sound insulation peak points (A_1_, B_1_, C_1_), the variation in effective dynamic mass is highly pronounced, and the large values render the structure acoustically rigid—characteristics that are representative of local resonant acoustic metamaterials.

Figure 2e shows the mode distribution of the sound insulation unit, while Figure 2f depicts the normal displacement of the center of the planar spring under an incident sound wave of 1 Pa. The valley frequencies A_0_, B_0_, and C_0_ in the STL curve approximately correspond to the resonant frequencies of the central-curved, single-curved, and double-curved structures, respectively. At these frequencies, the three curved structures exhibit significantly larger out-of-plane displacements. Specifically, at the valley points, the central cylindrical disk undergoes pronounced vertical oscillations in the same direction, driven by the excitation of the curved beams. The vibration directions of all points on the curved structures become highly aligned, whereas the remaining portions of the planar spring exhibit negligible displacement. This collective motion corresponds to a monopole mode, in which the sound insulation unit exhibits reduced insulation effectiveness, thereby producing sound insulation valleys.

In contrast, at the sound insulation peak frequencies A_1_, B_1_, and C_1_, the overall vibration amplitude of the sound insulation unit is relatively low. At peak A_1_, the displacement direction of the central cylindrical disk is opposite to that of the single-curved structure, forming a dipole mode. At sound insulation peak B_1_, the cylindrical disk of the single-curved structure vibrates in the opposite direction to that of the cylindrical disk of the double-curved structure. At sound insulation peak C_1_, the beam of the single-curved structure moves in opposition to its cylindrical disk, while the cylindrical disk of the double-curved structure vibrates in the same direction as that of the single-curved structure. Both B_1_ and C_1_ thus correspond to dipole sound radiation, which exhibits significantly lower sound radiation efficiency compared to the monopole mode, thereby contributing to the formation of sound insulation peaks.

### 3.2. Regulation of the Sound Insulation Band

The above analyses indicate that the resonant frequency of each curved structure plays an important role in shaping the peaks and valleys of the STL curve. Thus, by adjusting structural parameters—such as the length of the curved beams, the thickness of the planar spring, and the mass of the attached blocks—the dynamic properties can be regulated, thereby enabling regulation of the sound insulation band.

**Figure 3:**
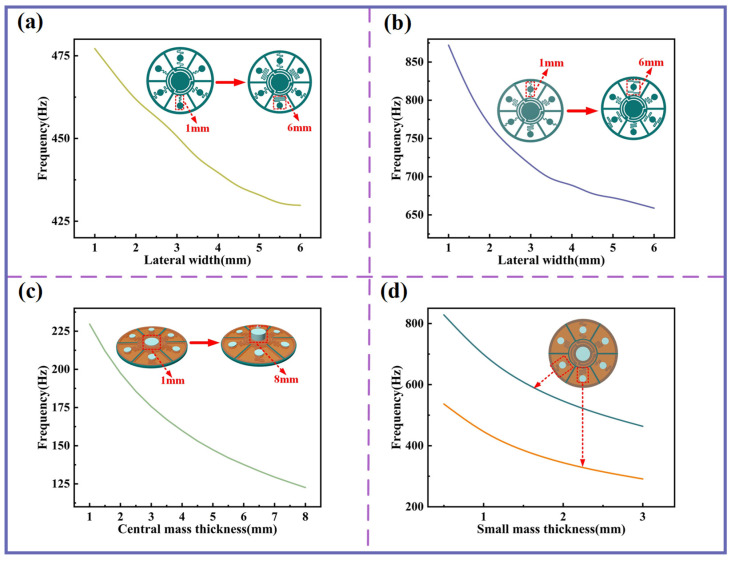
The influence of beam lateral width and mass blocks on resonant frequency. (**a**) Variation in resonant frequency of single-curved structures with different beam lateral widths. (**b**) Variation in resonant frequency of double-curved structures with different beam lateral widths. (**c**) Variation in resonant frequency with different central mass thicknesses. (**d**) Variation in resonant frequency with different small mass block thicknesses.

As shown in Figure 3a,b, increasing the lateral width of the curved beams in the single-curved and double-curved structures (equivalent to elongating the beam length) results in a gradual reduction in their resonant frequencies. Notably, for the double-curved structure, tuning the beam length exerts a particularly strong influence on its resonant behavior. Figure 3c demonstrates that increasing the thickness of the central mass block leads to a continuous decrease in the resonant frequency of the central-curved structure within the low-frequency regime. Similarly, Figure 3d shows that increasing the surrounding small proof mass thickness continuously reduces the resonant frequencies of both the single-curved and double-curved structures. Collectively, these approaches provide effective and versatile strategies for regulating resonant frequencies and tailoring the sound insulation performance of the device.

**Figure 4:**
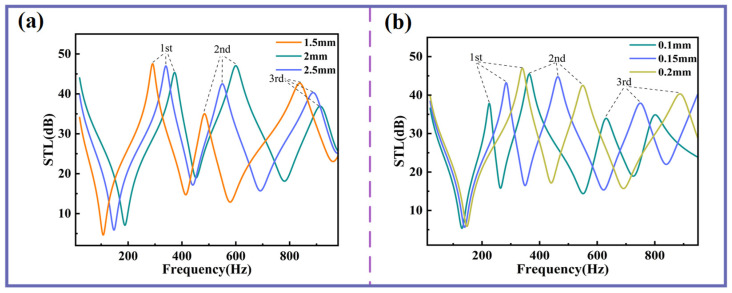
The influences of planar spring and Kapton thicknesses on STL. (**a**) STL curves with different flat spring thicknesses. (**b**) STL curves with different membrane thicknesses.

Figure 4a,b demonstrate that as the thickness of the planar spring or Kapton membrane increases, all three sound insulation peaks and valleys shift toward higher frequencies. From the perspectives of STL and sound insulation bandwidth, the thickness of the planar spring exerts a more pronounced influence on the second and third sound insulation peaks, significantly enhancing both their STL and the bandwidth of these peaks. In contrast, membrane thickness has a greater impact on the first sound insulation peak but exhibits almost no effect on the first sound insulation valley.

In practical applications, in confined space, adjusting the length or width of the curved beam is notably inconvenient. Meanwhile, modifying the size of the mass block to tune the resonant frequency of the curved structure yields only limited effectiveness; furthermore, an excessively large or heavy mass block contradicts the original objective of achieving a lightweight design. Therefore, from the standpoint of both tuning efficacy and practical feasibility, adjusting the thickness of planar spring and Kapton membrane constitutes a more effective approach for resonant frequency modulation.

**Figure 5:**
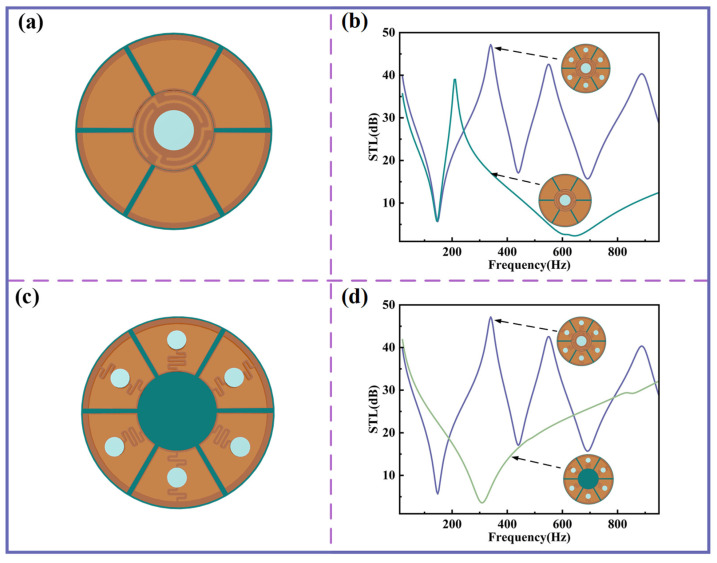
The influence of different curved structures on STL. (**a**) Schematic diagram for the case of a central-curved structure only. (**b**) STL comparison between various curved structures and only a central-curved structure. (**c**) Schematic diagram for the case without a central-curved structure. (**d**) STL comparison between various curved structures and without a central-curved structure.

The impact of planar spring structures on sound insulation performance is further examined. Figure 5a,b show the configuration and STL curves when only the central-curved structure and its surrounding Kapton membranes are retained. Under this condition, the first sound insulation valley nearly coincides with that of the original structure, while the second sound insulation peak is absent due to the lack of resonant contributions from the other curved structures. As illustrated in Figure 5c, replacing the bottom of the central-curved structure with a homogenous PLA disk yields the STL curve shown in Figure 5d, which demonstrates minimal sound insulation performance in the low-frequency range. These results confirm that the central-curved structure plays a pivotal role in achieving low-frequency sound insulation.

Table 2 summarizes the sound insulation performance under different structural conditions. It is evident that configurations incorporating curved structures consistently exhibit significantly enhanced insulation compared with the alternatives, thereby highlighting the distinct advantages of the proposed sound insulation unit.

### 3.3. Influence of Silicone Membrane on Sound Insulation Performance

**Figure 6:**
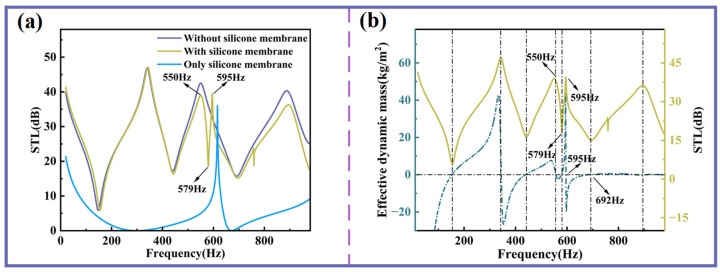
The influence of the silicone membrane on STL. (**a**) Sound insulation curves with and without silicone membrane. (**b**) Effective dynamic mass and STL curve with the silicone membrane.

Figure 6a compares the STL curves of three configurations: with the silicone membrane covering the sound insulation unit, without the silicone membrane, and with the silicone membrane alone. The silicone membrane by itself exhibits relatively weak insulation performance across most frequency bands, with only a single noticeable peak. When the silicone membrane is integrated into the unit, the first STL peak in the low-frequency range remains largely unaffected. However, above 400 Hz, a slight reduction in STL amplitude is observed. Notably, at 550 Hz, the STL decreases gradually, followed by a sharp increase at 579 Hz, giving rise to an additional insulation peak. This phenomenon can be attributed to the intrinsic resonance characteristics of the silicone membrane.

As shown in Figure 6b, it is evident that the zero points of the effective dynamic mass exhibit a strong correlation with the peaks and valleys of sound insulation. Notably, the frequency corresponding to the new sound insulation peak is remarkably close to the frequency at which the zero point of the effective dynamic mass occurs. In other words, at this new valley (579 Hz), there is a transition of the effective dynamic mass from negative to positive, resulting in a substantial increase in STL. Conversely, when the effective dynamic mass transitions from positive to negative at this same frequency, STL experiences a significant decrease. The other endpoint of this new sound insulation peak (692 Hz) corresponds to the resonant frequency of the double-curved structure and is independent of the silicone membrane.

## 4. Experimental Analysis

### 4.1. Acoustic Insulation Performance of MBSI-TENG

**Figure 7:**
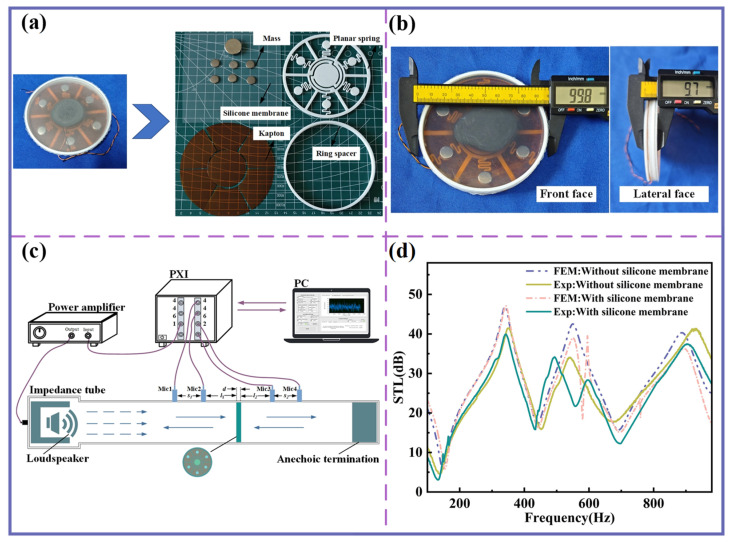
Components of MBSI-TENG and sound insulation evaluation. (**a**) Components of the MBSI-TENG. (**b**) Front and lateral views of the MBSI-TENG. (**c**) Schematic diagram of the impedance tube measurement for evaluating the sound insulation performance of the MBSI-TENG. (**d**) Comparison of FEM predictions and impedance tube experimental results.

A photograph of the fabricated components is shown in Figure 7a. The planar spring and ring spacer were manufactured using a 3D printer (Bamboo A1) based on Fused Deposition Modeling (FDM) technology, with PLA as the raw material. The Kapton membranes were pre-cut into seven individual pieces using a laser cutter, and all components were assembled with glue. Figure 7b presents the front and side dimensional diagrams of the MBSI-TENG.

To evaluate the sound insulation performance, a 100 mm diameter impedance tube was employed following the ASTM E2611-09 standard (Figure 7c). A PXI-4461 module generated a band-limited white noise signal, which was then power-amplified and delivered to a loudspeaker inside the tube. Four 1/4-inch microphones (model: G.R.A.S 40PH, 50 mV/Pa) were positioned to measure sound pressure before and after transmission through the MBSI-TENG, and a PXI-4462 module was used for signal acquisition.

The experimental results are shown in Figure 7d. The STL curves exhibit multiple distinct insulation peaks and valleys, consistent with the FEM predictions, both with and without the silicone membrane. The measured average sound insulation within 1000 Hz exceeds 25 dB. A frequency shift is observed in both cases, particularly with the silicone membrane. This shift is attributed to the complex nonlinear acoustic–vibration coupling among the silicone membrane, the sound insulation unit, and the trapped air layer.

### 4.2. Acoustic–Electric Conversion Performance of MBSI-TENG

**Figure 8:**
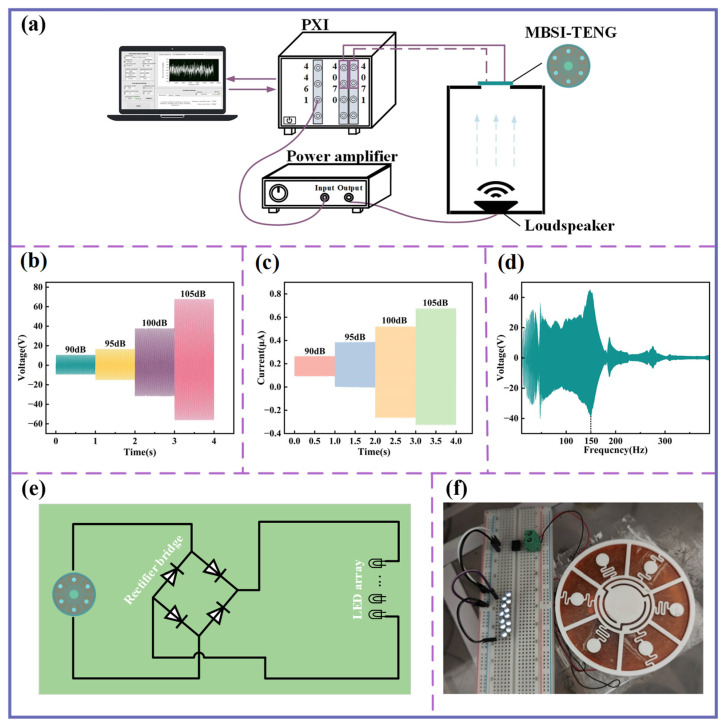
Acoustic–electric conversion performance of the MBSI-TENG. (**a**) Experimental setup for evaluating the acoustic-to-electric conversion performance of the MBSI-TENG. (**b**) Voltage signals under different SPL values. (**c**) Current signals under different SPL values. (**d**) Voltage response under swept sine excitation. (**e**) Circuit diagram for MBSI-TENG acoustic-to-electric conversion. (**f**) Photograph showing the LED array being powered.

As demonstrated in the preceding analysis, the MBSI-TENG exhibits local resonance characteristics near the sound insulation valleys, with the central-curved structure undergoing significant displacement at the first valley frequency. Figure 8a presents the experimental setup for Acoustic–Electric Conversion. The output voltage of the MBSI-TENG was measured using a high-performance digital multimeter (PXI-4070) connected through a 100× attenuation probe to optimize input impedance and provide high-voltage protection. The output current was measured with a PXI-4071 module. The device was mounted at the open top of a 20 mm-thick acrylic enclosure, with all joints sealed to minimize acoustic leakage.

Figure 8b,c show the peak-to-peak voltage and short-circuit current of the MBSI-TENG as the excitation SPL increased from 90 dB to 105 dB. Both quantities increase significantly with rising SPL, confirming the device’s ability in acoustic-to-electric conversion. Figure 8d illustrates the voltage response under sinusoidal sweep excitation, where a broad operational frequency range is observed. The response reaches its maximum at 150 Hz, which is close to the resonant frequency of the central-curved structure, in good agreement with design expectations.

The practical applicability of the MBSI-TENG was further demonstrated by integrating it with a bridge rectifier circuit (Figure 8e) to drive an LED array. As shown in Figure 8f, the device successfully powered the LEDs under acoustic excitation, highlighting its superior acoustic-to-electric conversion capability and strong potential for real-world applications.

**Figure 9:**
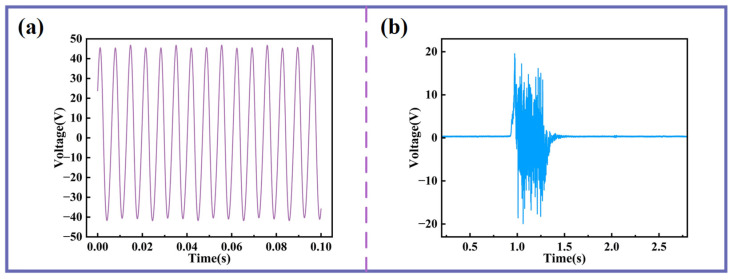
The application potential of MBSI-TENG in acoustic sensing. (**a**) Voltage response under 150 Hz sinusoidal excitation. (**b**) Voltage response during gentle blowing.

Additionally, the MBSI-TENG demonstrates significant potential for acoustic sensing applications. When a sinusoidal acoustic excitation is applied, the measured voltage signal from the MBSI-TENG is shown in Figure 9a. The output closely follows an ideal sinusoidal profile with clear periodicity. In addition, as shown in Figure 9b, when a gentle airflow—achieved by softly blowing air onto the silicone membrane—is applied, the voltage amplitude increases instantaneously, reaching a peak-to-peak value of nearly 40 V. These results further emphasize the high sensitivity of the MBSI-TENG to external acoustic stimuli.

## 5. Conclusions

In this work, we propose a novel multifunctional MBSI-TENG that integrates multiple local resonances design, achieving efficient sound insulation across broad frequency bands while simultaneously enabling low-frequency acoustic-to-electric conversion. The mechanisms underlying multi-band sound insulation and the advantages of different curved structures were systematically investigated and elucidated. The sound transmission loss curves obtained from impedance tube experiments align well with FEM predictions. The sound insulation unit achieves a maximum STL of 40 dB and maintains an average STL above 25 dB within the 1000 Hz range, demonstrating exceptional broadband sound insulation performance.

Furthermore, by incorporating an elastic silicone membrane, the MBSI-TENG effectively enables acoustic-to-electric conversion at low frequencies while retaining its sound insulation capabilities, allowing it to power small-scale electronic devices. Looking ahead, the MBSI-TENG shows strong potential for deployment in practical industrial environments and offers broad prospects for future applications in multifunctional acoustic systems.

## Figures and Tables

**Table 1:** Material parameters of the MBSI-TENG.

Material	Young’s Modulus (MPa)	Density (kg/m^3^)	Poisson’s Ratio
PLA	2530	1240	0.38
Kapton	2760	1420	0.34
Steel	20,000	8356	0.28
Silicone membrane	0.5	1200	0.48

**Table 2:** The Average sound transmission loss of three different sound insulation unit structures.

Type	Various Curved Structures	Without a Central-Curved Structure	Only a Central-Curved Structure
Average STL(dB)	28.236	22.428	13.031

## Data Availability

Data will be made available on request.

## Abbreviations

The following abbreviations are used in this manuscript:

TENG	Triboelectric Nanogenerator
MWCNTs	Multi-walled Carbon Nanotubes
MAM	Membrane-type Acoustic Metamaterial
IoT	Internet of Things
MBSI-TENG	Multi-Band Sound Insulation Triboelectric Nanogenerator
PLA	Polylactic Acid
PI	Polyimide
FEP	Fluorinated Ethylene Propylene
FEM	Finite Element Method
STL	Sound Transmission Loss
SPL	Sound Pressure Level
FDM	Fused Deposition Modeling
